# RETRACTED: Zhang et al. Shikonin Inhibits the Migration and Invasion of Human Glioblastoma Cells by Targeting Phosphorylated β-Catenin and Phosphorylated PI3K/Akt: A Potential Mechanism for the Anti-Glioma Efficacy of a Traditional Chinese Herbal Medicine. *Int. J. Mol. Sci.* 2015, *16*, 23823–23848

**DOI:** 10.3390/ijms26135922

**Published:** 2025-06-20

**Authors:** Feng-Ying Zhang, Yi Hu, Zhong-You Que, Ping Wang, Yun-Hui Liu, Zhen-Hua Wang, Yi-Xue Xue

**Affiliations:** 1 Department of Neurobiology, College of Basic Medicine, China Medical University, Shenyang 110122, China; zhangfengyingcmu@163.com (F.-Y.Z.); pingwang8000@163.com (P.W.); 2 Institute of Pathology and Pathophysiology, China Medical University, Shenyang 110122, China; 3 Department of Neurosurgery, Shengjing Hospital of China Medical University, Shenyang 110004, China; hooyie@hotmail.com (Y.H.); 18840042401@163.com (Z.-Y.Q.); liuyunhuicmu@aliyun.com (Y.-H.L.); 4 Department of Physiology, College of Basic Medicine, China Medical University, Shenyang 110122, China; hooyie@sohu.com

The journal retracts the article titled “Shikonin Inhibits the Migration and Invasion of Human Glioblastoma Cells by Targeting Phosphorylated β-Catenin and Phosphorylated PI3K/Akt: A Potential Mechanism for the Anti-Glioma Efficacy of a Traditional Chinese Herbal Medicine” [1], cited above.

Following publication, concerns were brought to the attention of the Editorial Office regarding the integrity of a number of images presented in this article [1].

Adhering to our standard procedure, the Editorial Office and Editorial Board conducted an investigation that confirmed image modification and duplication between Figure 2A,B published in [1], within Figure 8 in [1], and between Figures 2 and 3 in [1] and Figures 2C and 5B in [2], presenting different experimental conditions. The authors were contacted on multiple occasions but failed to respond. Consequently, the Editorial Board has lost confidence in the reliability of the findings and decided to retract this publication [1] as per MDPI’s retraction policy (https://www.mdpi.com/ethics#_bookmark30).

This retraction was approved by the Editor-in-Chief of the *International Journal of Molecular Science*.

The authors did not provide a comment on this decision.
